# Plasma Levels of Free *N^Ɛ^*-Carboxymethyllysine (CML) after Different Oral Doses of CML in Rats and after the Intake of Different Breakfasts in Humans: Postprandial Plasma Level of sRAGE in Humans

**DOI:** 10.3390/nu14091890

**Published:** 2022-04-30

**Authors:** Cynthia Helou, Matheus Thomaz Nogueira Silva Lima, Céline Niquet-Leridon, Philippe Jacolot, Eric Boulanger, Florian Delguste, Axel Guilbaud, Michael Genin, Pauline M. Anton, Carine Delayre-Orthez, Tatiana Papazian, Michael Howsam, Frédéric J. Tessier

**Affiliations:** 1 Department of Nutrition, Faculty of Pharmacy, Saint Joseph University of Beirut, Beirut 1004 2020, Lebanon; cynthia.helou@usj.edu.lb (C.H.); tatiana.wahanian@usj.edu.lb (T.P.); 2 U1167—RID—AGE—Facteurs de Risque et Déterminants Moléculaires des Maladies Liées au Vieillissement, Institut Pasteur de Lille, University Lille, Inserm, CHU Lille, F-59000 Lille, France; matheusthomaz.nogueirasilvalima@univ-lille.fr (M.T.N.S.L.); eric.boulanger@univ-lille.fr (E.B.); florian.delguste@gmail.com (F.D.); axel.guilbaud@gmail.com (A.G.); michael.howsam@univ-lille.fr (M.H.); 3 ULR 7519, Equipe PETALES, Institut Polytechnique UniLaSalle, Université d’Artois, F-60026 Beauvais, France; celine.leridon@unilasalle.fr (C.N.-L.); philipe.jacolot@unilasalle.fr (P.J.); pauline.anton@unilasalle.fr (P.M.A.); carine.delayre@unilasalle.fr (C.D.-O.); 4 ULR 2694-METRICS: Évaluation des Technologies de Santé et des Pratiques Médicales, University Lille, Inserm, CHU Lille, F-59000 Lille, France; michael.genin@univ-lille.fr

**Keywords:** glycation, Maillard reaction, carboxymethyllysine, lysine, sRAGE

## Abstract

N-carboxymethyl-lysine (CML) and other dietary advanced glycation end-products (AGEs) are chemically modified amino acids with potential toxicological effects putatively related to their affinity with the receptor for AGEs (RAGE). The goal of this study was to determine the postprandial kinetics of CML in both rodents and humans and, in the latter, to evaluate their relationship with the soluble RAGE isoforms (sRAGE). Four gavage solutions containing different forms of CML were given to rats, and blood was collected over 8 h. Three different breakfasts containing dietary CML (dCML) were administered to 20 healthy volunteers, and blood was collected over 2 h. Concentrations of CML, CEL, and lysine were quantified in plasma and human meals by LC-MS/MS, and sRAGE was determined in human plasma by ELISA. The results showed that dCML did not affect the concentrations of circulating protein-bound CML and that only free CML increased in plasma, with a postprandial peak at 90 to 120 min. In humans, the postprandial plasmatic sRAGE concentration decreased independently of the dAGE content of the breakfasts. This study confirms reports of the inverse postprandial relationship between plasmatic free CML and sRAGE, though this requires further investigation for causality to be established.

## 1. Introduction

Chemical reactions occur between the constituents of foods during domestic and industrial processing, as well as during storage. While these reactions can have beneficial effects on the aroma, taste, and appearance of foods, they are also responsible for the degradation of some nutrients [1].

When proteins are involved, one of the main chemical reactions responsible for modifying the side chain of some amino acids is the Maillard reaction, also referred to as “glycation”. This reaction leads to the covalent attachment of reducing sugars and other carbonyl and dicarbonyl compounds to some reactive amino acids such as lysine and arginine [2].

Advanced glycation end-products in the diet (dAGEs) are among the heterogeneous group of Maillard reaction products (MRPs) formed by chemical rearrangements which follow the initial binding of the sugar moiety to amino acids, some of which result in organoleptic improvements of food while others are widely studied for their potential negative impacts on health [2,3].

Most dAGEs found in foodstuffs are non-physiological amino acids that cannot be used as sources of amino acids for protein anabolism in vivo. In addition to this reduction of dietary amino acid availability and, more generally, to the impairment of the nutritional quality of heated proteins, the metabolic transit and the potential toxicological effects of dAGEs have been subjects of concern for more than 4 decades [4,5].

N-carboxymethyl-lysine (CML) was the first dAGE identified in food, human tissues, and urine [5]. The precise rate of its absorption into the bloodstream, the subsequent distribution and clearance of dietary CML (dCML) from different foods remain ill-defined; however, despite several recent studies of its bioavailability and postprandial kinetics. dCML is largely studied not only because it is considered a good model for the metabolic transit and biological effects of dAGEs, but also because of its relatively high abundance in foods. Different food groups have been analyzed to estimate their contribution to dCML intake, and it has been reported that bakery products are among its major sources [3,6].

Using an in vitro model of human intestinal absorption, it has been found that dAGEs are absorbed in their dipeptide form, presumably via the intestinal peptide co-transporter PepT1 [7]. After intracellular hydrolysis of peptide bonds, free glycated amino acids such as dCML were reportedly released into the in vitro equivalent to the systemic circulation.

This observation agrees with our preliminary data on the postprandial changes in plasma CML concentrations in rats. After an oral intake of food rich in dCML, free CML increased quickly in plasma, whereas protein-bound CML remained unaffected by the dCML intake [8].

Here, we build upon this earlier study and contribute to the understanding of the postprandial kinetics of dCML in both rodents and humans. We measured the changes in plasma CML in rats after receiving four different forms of dCML and in healthy human volunteers after receiving three different breakfasts. In addition, in the human plasma samples, we also measured the changes in soluble RAGE (sRAGE) concentrations, the Receptor for Advanced Glycation End-products being widely considered to be that with which AGEs principally interact.

## 2. Materials and Methods

### 2.1. Chemicals and Materials

Ultra-pure HPLC water was from VWR (Fontenay-sous-Bois, France), HPLC gradient grade acetonitrile, nonafluoropentanoic acid (NFPA) 97%, hydrochloric acid (HCl) 37%, sodium borohydride (NaBH_4_), boric acid (H_3_BO_3_) and sodium hydroxide (NaOH), trichloroacetic acid (TCA), lysine (Lys), bovine serum albumin (BSA fraction V), glyoxylic acid and sodium cyanoborohydride (NaBH_3_CN) were all obtained from Sigma–Aldrich (Saint-Quentin-Fallavier, France). The labeled internal standard (^15^N_2_)-Lys was purchased from CortecNet (Voisins-le-Bretonneux, France), while CML, (D2)-CML, (D4)-CML, CEL, and (D4)-CEL were all from Polypeptide Group Laboratories (Strasbourg, France). A 0.1 M NaBH_4_ solution was made up of a 0.2 M borate buffer comprised of (H_3_BO_3_) and NaOH (pH = 9.5).

### 2.2. Preparation of the CML-Enriched BSA (CML-BSA)

CML-enriched bovine serum albumin (CML-BSA) was prepared as previously described [9]. Briefly, BSA faction V was incubated with glyoxylic acid (60 mM) in phosphate buffer (200 mM, pH 7.4) at 37 °C for 20 h. After 2 h of incubation, NaBH_3_CN (450 mM) was added to the solution. After incubation, the preparation was dialyzed at 4 °C against phosphate buffer (200 mM, pH 7.4). The dialyzed preparation was lyophilized, and the dried powder was stored in an air-tight container at −20 °C until analysis and utilization in the animal study. The CML-BSA contained 78.1 ± 4.0 mg CML/g BSA.

### 2.3. Single Oral Dose Studies of Free CML, Free (D2)-CML, CML-BSA, and Free CML + BSA in Rats

One hundred and twenty male Wistar rats (200–224 g–8 weeks old) from Harlan Laboratories (Ganat, France) were housed in stainless steel cages under controlled temperature (21 ± 1 °C) and a 12 h light-dark cycle. During the first week (adaptation period), all rats received A04 pellets (Safe, Augy, France) and water *ad libitum*. At the end of the adaptation period, rats were divided into four groups of 30. The first group received a single oral dose of free CML solution (410 µg/rat; 1.48 mg/kg BW); the second a dose of free D_2_-CML solution (520 µg/rat; 1.78 mg/kg BW); the third a dose of CML-BSA solution (415 µg/rat; 1.43 mg/kg BW); and the last group received a dose of the free CML + BSA solution mix (491 µg/rat; 1.60 mg/kg BW). Six rats per group were sacrificed 0, 1, 2, 5, and 8 h after gavage, and blood (2 mL/animal) was collected. At the given time point, rats were anesthetized with an intraperitoneal injection of a mixture of ketamine and xylazine (50% *v*/*v*), 100 mg/mL) diluted in saline at a dose of 1 mL/kg. This experiment was conducted at the animal house unit of the Institut Polytechnique LaSalle Beauvais (LaSalle facilities agreement number C60-200-001,) and received prior approval from both the local animal protocol review committee (CEEA n°116) and the French Ministry of Education (MENESR, n°03530.V02).

### 2.4. Human Intervention Study

#### 2.4.1. Study Design and Participants

In this longitudinal crossover study, twenty healthy individuals received three test meals with a 7-day washout period in-between. The test meals were breakfasts inspired by Mediterranean and Westernized diets and included a regular and a grilled version of the latter. For practical reasons, all participants received the breakfasts in the same order, and participants were also kept ignorant of the meals’ contents until consumption.

Participants were recruited according to the following inclusion criteria: age between 18 and 30 years, a body mass index (BMI) between 20 and 25 kg/m^2^, a waist circumference below 80 cm for women and 94 cm for men, a systolic blood pressure ≤ 130 mm Hg and diastolic blood pressure ≤ 85 mm Hg, and normal fasting plasma glucose, triglycerides and cholesterol levels. Subjects were considered ineligible if they suffered from a chronic disease (e.g., type 1 or 2 diabetes, cancer, cardiovascular disease), were smokers, used medication regularly, reported more than 4 h of physical activity per week, had bariatric surgery, or, for female participants, were pregnant or breastfeeding.

During the screening visit, anthropometric measurements (weight, height, waist circumference, body fat composition) and blood pressure were recorded. A medical history questionnaire, as well as a food frequency questionnaire and a 24-h dietary recall, were completed by the research team during a face-to-face interview. Prior to enrollment, all selected participants gave written, informed consent. The protocol of this study was approved by the ethical committee of Saint-Joseph University of Beirut, Lebanon (FPH58/2017).

#### 2.4.2. Study Protocol

Throughout the study period, participants were instructed not to consume fried, grilled, or roasted foods and to refrain from intense physical activity and alcohol consumption. On the evenings before the test days, they were requested to consume their dinner no later than 20h00, though water intake was permitted until midnight.

Subjects arrived on the test days with minimal physical effort. A blood sample was drawn at baseline (0 min) to assess the fasting levels of the studied biochemical markers. Then, the participants had 15 min to consume the test meals. Three other blood samples were then taken at 45, 90, and 120 min following the first bite from the test meals.

#### 2.4.3. Test Meals

The three meals assessed during this study had similar caloric values but differed in macronutrient content. Methods corresponding to the analysis of caloric content, fat, carbohydrates, protein, ash, moisture, minerals, and vitamins in each tested breakfast are properly indicated in Supplemental Table S1. The Mediterranean-inspired breakfast (MB) consisted of 65 g whole wheat pita bread (Moulin d’Or, Beirut, Lebanon) with 57 g feta cheese (Dodoni, Greece) and 16 g black olives purchased from a local supermarket. The Western-inspired breakfast (WB) consisted of 57 g of Emmental cheese (Zott, Mertingen, Germany) with turkey ham (Reinert, Versmold, Germany) sandwiched in 79 g of soft white bread (Moulin d’Or, Lebanon). The third meal, the Grilled Western-inspired Breakfast (GWB), was identical to the WB in composition, the difference between WB and GWB being that the sandwich was grilled for 4 min using a household grill (Russel Hobbs, 1960 W, Sulzbach, Germany). Identical quantities of water (180 mL), cucumbers (80 g), cherry tomatoes (50 g), and apples (130 g) were provided with all three breakfasts. Detailed nutrient compositions are presented in Table 1.

### 2.5. CML Quantification in Plasma from Rats

Total and protein-bound CML were analyzed in plasma samples from rats, both fractions undergoing a reduction step prior to acid hydrolysis. After centrifugation, 25 µL of plasma were placed in a pyrex tube with 100 µL ultra-pure water and 500 µL NaBH_4_ 0.1 M for 2 h at ambient temperature. For total CML analysis, 625 µL of 12 M HCl were added, and the mixture was incubated for 20 h at 110 °C in an oven. For protein-bound CML analysis, a protein precipitation step after reduction was first performed by adding 2 mL of TCA 200 g/L. After centrifugation (2000× *g* 10 min), the supernatant was removed. Then, 500 µL HCl 6 M was added, and the tube was incubated for 20 h at 110 °C. An aliquot of each hydrolysate (200 µL) was reduced to dryness in a Speedvac concentrator (ThermoFisher Scientific, Courtaboeuf, France) and stored at −80 °C until analysis.

Liquid chromatography with tandem mass spectrometry (LC-MS/MS) analyses of plasma samples from rats were carried out on a TSQ Quantum Ultra (ThermoFisher) with a Heated Electrospray Ionization (HESI) probe coupled to an Accela HPLC system (ThermoFisher). The chromatographic separation was performed on a Hypercarb column (100 × 2.1 mm, 5 µm, ThermoFisher) with a guard column of the same phase. The column was maintained at 10 °C, and the injection volume was 20 µL. The elution was performed with aqueous 20 mM NFPA solution (solvent A) and acetonitrile (solvent B) at a flow rate of 200 µL/min with the following gradient: percentage of B: 0–10 min, 0–15%; 10–15 min, 15–20%; 15–18 min, 20–50%; 18–22 min, 50%; 22–23, 50–0%; 23–28 min, 100%.

The tandem MS analyses measured the following specific transitions (in elution order): *m*/*z* 147.0 → 130.0 and *m*/*z* 149.0 → 131.0 for lysine and its isotope, respectively; *m*/*z* 205.0 → 130.0, 207.0 → 130.0 and *m*/*z* 209.0 → 134.0 for CML and its D2 and D4 isotopes, respectively; *m*/*z* 219.0 → 130.0 and *m*/*z* 223.0 → 134.0 for CEL and its isotope, respectively. Quantification of all compounds utilized the ratio between the peak areas of the analyte: peak area of its isotope (internal standard) and comparison with 7-point calibration curves.

Free CML concentration was obtained by subtracting the protein-bound CML concentration from the total CML concentration.

### 2.6. CML Quantification in the 3 Human Breakfasts and Human Plasma

The LC-MS/MS analyses were performed on a Vantage instrument with an HESI source (ThermoFisher). A Hypercarb column (100 × 2.1 mm, 5µm; ThermoFisher) with a guard column (10 × 2.1 mm, 5 µm, same phase) was used for chromatographic separation (column temperature 10 °C). Binary mobile phase flow rate was 200 µL/min (A–aqueous 10 mM NFPA, B–acetonitrile; percentage of B: 0–9 min, 0–25%; 9–11 min, 25–60%; 11–13 min, 60%; 13.1–21 min, 0%). The tandem MS analyses measured the same transitions as above (though without D4-CML), and quantification of all compounds utilized the analyte: isotope (internal standard) ratios and comparison with 9-point calibration curves.

A well-mixed, representative aliquot of each breakfast was lyophilized, and three sub-samples of each were analyzed by LC-MS/MS. The ca. 150 mg sub-samples were resuspended in 100 µL Ultra-pure HPLC water, to which 500 µL of 0.1 M NaBH_4_ was added (2 h at ambient temperature) to reduce the samples and prevent de novo production of CML during subsequent acid hydrolysis. Then, 600 µL of 12 M HCl was added, and the sample was hydrolyzed for 21 h at 110 °C. An aliquot (200 µL) was reduced to dryness in a Speedvac concentrator and stored at −80 °C until analysis.

Plasma samples were first subjected to a protein precipitation procedure: 50 µL of plasma was vortexed with 25 µL of aqueous 10% TCA and proteins were left to precipitate on ice for 30 min. Samples were centrifuged at 21,000× *g*, 4 °C for 10 min, and 30 µL of the supernatant (representing nearly all that could be taken and containing the “free” CML), was aspirated and reduced to dryness in a Speedvac, as above. Because the main objective of our study was to follow the metabolic fate of dCML, and because its impact on protein-bound CML is non-existent, it was decided to study only plasmatic-free CML in the human intervention study.

Immediately before analysis, the breakfast samples were thawed and diluted 20× with aqueous 12.5 mM NFPA containing (D2)-CML and (D4)-CEL, and a further 10× serial dilution with NFPA and (^15^N_2_)-Lys was prepared for the quantification of this amino acid. Plasma samples were thawed and resolubilized in 30 µL of 12.5 mM NFPA containing (D2)-CML, (D4)-CEL, and (^15^N_2_)-Lys. The preparation and analysis of samples were randomized to avoid any bias from interday variations in instrument performance.

### 2.7. sRAGE Quantification in Human Plasma

Soluble RAGE (sRAGE) was quantified in duplicate 50 μL aliquots of human plasma for each time point from all individuals by ELISA (Quantikine DRG00, R & D Systems, Oxford, UK), following the manufacturer’s instructions. This ELISA was able to detect 2 isoforms of sRAGE: the endogenous secretory (esRAGE) and the cleaved (cRAGE) isoforms. Ninety-six-well plates were read on a Fluorostar Omega plate reader (BNG Labtech, Champigny sur Marne, France), and samples were randomized among the different plates. In fact, calibration curves’ gradients among the plates varied by less than 10%, while results for samples quantified on several plates were within 1% (data not shown).

### 2.8. Statistical Analyses

Differences in free CML levels and their iAUC in rat samples, and in free CML and CEL, free lysine, and sRAGE levels and their iAUC in human plasma samples, as well as differences in nutritional composition among the different meals presented in Table 1, were all analyzed using ANOVA followed by Tukey’s post hoc test for pairwise comparisons. iAUC values were calculated using the trapezoidal rule after baseline correction. Gender differences were evaluated by the student’s two-tailed *t*-test. The correlations between the different parameters were evaluated using Pearson’s correlation coefficient. All data are presented as means +/− Standard Deviation (SD). Statistical analysis was performed using GraphPad Prism 9.0 (San Diego, CA, USA) or Minitab 19 using α = 0.05 as a threshold value for statistical significance.

## 3. Results

### 3.1. Kinetics of Free and Protein-Bound CML in Rat Plasma after Gavage with Four Different Forms of CML

The four gavage solutions contained similar quantities of CML, and rats received 410 to 520 µg CML, corresponding to 1.43 to 1.78 mg of CML per kg of body weight, in a single oral dose. As a comparison, the maximum human exposure to dCML is estimated at 1.1 mg/kg BW/day [10]. The only difference between the four gavages was the form of CML in the solutions. The animals received either free CML, a free deuterated isotope of CML ((D2)-CML), bovine serum albumin-bound CML (CML-BSA), or a mix of free CML and BSA (CML + BSA).

**Figure 1:**
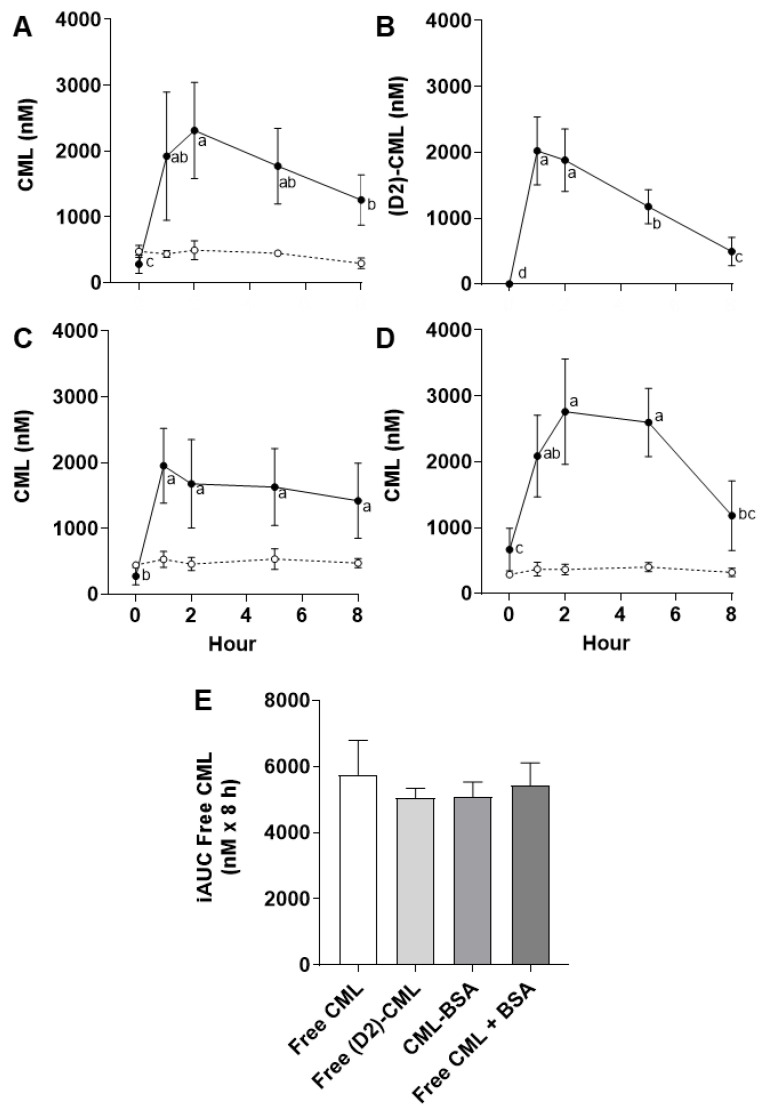
Kinetics of free (●) and protein-bound CML (○) in rats performed over 8 h after a single oral dose of (**A**) free CML, (**B**) free (D2)-CML, (**C**) protein-bound CML (CML-BSA), and (**D**) free CML mixed with BSA (CML + BSA). Charts shows mean ± SD values of CML concentration over time. Different letters denote statistical differences among different time points within free CML measures, taking *p* < 0.05 to be significant. (**E**) shows iAUC_Free CML 0–8 h_ for (**A**–**D**). Bars represent mean ± SD values. No statistical significance was observed (*p* < 0.05).

The mean fasting plasma concentrations of free and protein-bound CML before gavage were 437 ± 283 (*n* = 18) and 391 ± 93 (*n* = 18) nM, respectively (Figure 1A,C,D). This corresponds to what we had previously observed in male rats of identical age and genotype [8].

After free CML was administered, its plasma concentration increased to a maximum of 2316 ± 732 nM after 2 h, then decreased slowly to 1259 ± 384 nM at the end of the experiment (8 h) (Figure 1A). Since a linear elimination of free CML was observed from 2 to 8 h, it can be postulated that the rate of decrease in plasma CML concentration depends solely upon a rate constant (slope) and follows a zero-order kinetic model. Using the calculated linear equation, *[plasma free CML]* = −176 *t* + 2664, (R^2^ = 0.999), it can be estimated that it would take approximately 15 h to return to the fasting concentration of free CML in plasma. In addition, the time required to eliminate 50% of the maximal free CML concentration is estimated to be 7 h (t_1/2_ = 2316/(2 × 176)). The protein-bound concentration of CML in plasma of free-CML challenged rats remained stable throughout the experiment (431 ± 110 nM) (Figure 1A). This observation confirms that the systemic concentration of protein-bound CML is not affected by a single oral intake of free CML.

A dose of free (D2)-CML was compared with the gavage of free native CML above to verify the specificity of plasma-free CML measurements. Free CML in plasma has a dual origin: exogenous from the digestion of dietary glycated proteins and endogenous from the catabolism of in vivo glycated proteins. The use of an isotope of CML as a source of dCML, combined with quantification by LC-MS/MS, is the most accurate way to follow the metabolic transit of this glycation product and avoids potential confusion with endogenous sources of free CML. The postprandial kinetics of free (D2)-CML in plasma are shown in Figure 1B, and it can be seen that the same pattern was observed with free native CML (Figure 1A). The concentration of free (D2)-CML increased as steeply as free native CML, also reached a maximum at 2 h, and its elimination also followed the same trend as that for free native CML. The linear equation of free (D2)-CML elimination, *[plasma free (D2)-CML]* = −233 *t* + 2362, (R^2^ = 0.999), also shows a similar rate constant to that calculated for the elimination of free native CML. Using this last equation, we estimate a total elimination of free (D2)-CML at 10 h post-gavage and a t_1/2_ of 5 h.

Overall, then, the postprandial kinetics of free (D2)-CML overlap almost perfectly with free native CML, the two approaches differing only in their baseline and maximal plasma concentrations (Figure 1A,B).

After a single oral dose of protein-bound CML (CML-BSA), the postprandial concentration of free CML in plasma (Figure 1C) followed a different pattern than that observed with free CML and free (D2)-CML. After a marked increase during the first hour, the plasma concentrations of free CML remained relatively high for the duration of the experiment. Clearance of this protein-bound dCML appeared to be much slower when compared to the elimination of free CML or free (D2)-CML. The mean protein-bound CML concentration measured in plasma was again stable over the experimental period (490 ± 105 nM) and apparently not influenced by the gavage of CML-BSA (Figure 1C).

Lastly, a fourth form of dCML was tested (Figure 1D). In this case, free CML was mixed with non-glycated BSA, and the protein in this formulation affected the kinetics of free CML transfer into the circulation. Figure 1D shows a unique kinetic profile compared with the other three (Figure 1A–C). Notably, after consumption of the CML + BSA mix, plasma-free CML increased rapidly from 0 to 2 h, reached a plateau from 2 to 5 h, and then decreased more rapidly from 5 to 8 h than any of the other doses tested (slope = −472). The mean concentrations of protein-bound CML in plasma at baseline and after feeding with the mix were similar to each other (348 ± 80 nM) and also to those measured in the other groups of animals.

There was no significant difference among the four forms of dCML gavage for the postprandial Incremental Area Under the Curve (iAUC_0–8 h_) plasmatic free CML response (Figure 1E), despite the different kinetic profiles observed.

### 3.2. Comparative Interventional Study

#### 3.2.1. Composition of the Three Breakfasts

The mean caloric contents of the three breakfast meals were not significantly different (*p* = 0.912). MB, WB, and GWB provided 482.5 ± 50.2, 458.9 ± 28.6, and 471.5 ± 34.7 kcal/meal, respectively. However, the three meals differed in macronutrient content, as presented in Table 1. Total CML and CEL concentrations were only significantly higher in the GWB compared to the 2 other breakfasts (0.66 ± 0.01 and 0.82 ± 0.02 mg/100 g of the meal, respectively) (*p* < 0.05). When adjusted to the mean total intake of breakfast, volunteers received an average of 2.02 ± 0.05, 2.03 ± 0.03 and 2.73 ± 0.04 mg CML, and 1.83 ± 0.05, 1.66 ± 0.03, and 3.39 ± 0.05 mg CEL, from the MB, WB, and GWB respectively. For CML, it corresponded to dietary exposure of 0.03 to 0.04 mg of CML per kg of body weight. The total intake of dietary lysine was dependent on the protein quality and quantity of each breakfast. Thus, the total quantity of lysine varied from 1836 ± 43 mg/meal in the MB to 2817 ± 46 mg/meal in the GWB.

#### 3.2.2. Free CML, CEL, Free Lysine, and sRAGE at Baseline in Human Plasma

**Figure 2:**
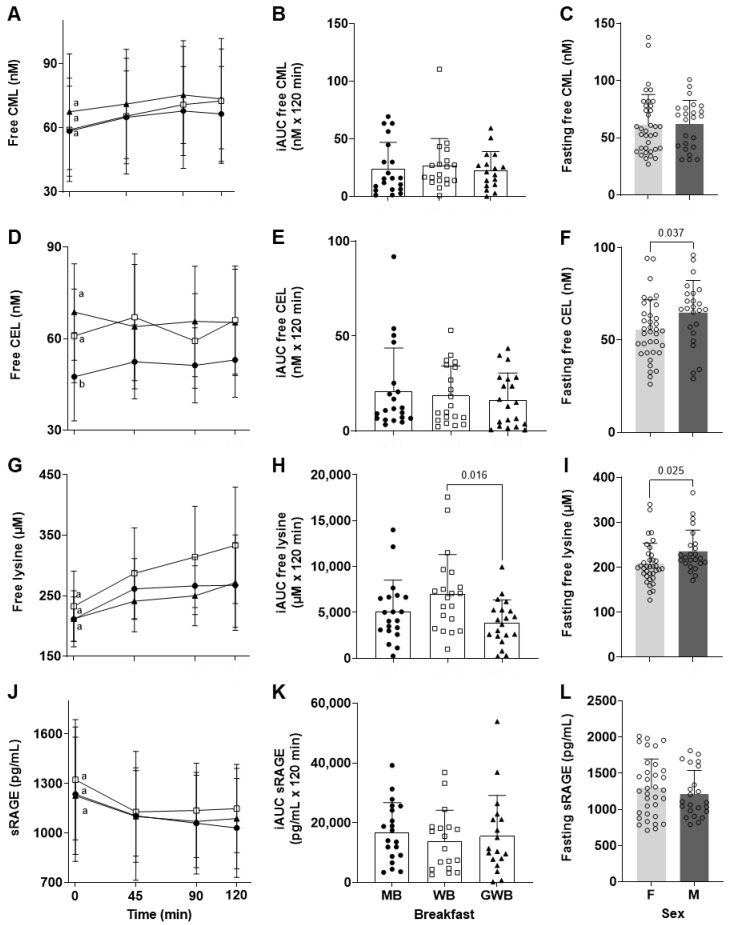
Circulating levels of free AGEs, free lysine, and sRAGE in human plasma. Kinetics of (**A**) free CML, (**D**) free CEL, (**G**) free lysine; and (**J**) sRAGE were compiled from four plasma samples 0 min (fasting), 45, 90, and 120 min after intake of the MB (●), WB (□), and GWB (▲) breakfasts. Letters denote statistical similarity among the different fasting measures prior to breakfast consumption (*p* < 0.05). The iAUC_0–120 min_ of each breakfast is shown for (**B**) free CML, (**E**) free CEL, (**H**) free lysine; and (**K**) sRAGE levels. Gender effects for fasting (**C**) free CML, (**F**) free CEL, (**I**) free lysine; and (**L**) sRAGE levels are shown for the pool of female (**F**) and male (M) volunteers over the 3 interventions (*n* = 60). For both the iAUC_0–120 min_ and gender analyses, statistically significant differences are indicated with significant *p* values at the level α = 0.05. Data represent mean ± SD.

The design of the interventional study included the collection of fasting blood samples on 3 consecutive weeks, with a 7-day washout period in between, in which free CML, CEL, and lysine were quantified by LC-MS/MS, and sRAGE by ELISA. In Figure 2A,D,G,J, the data presented at time 0 of the three kinetic studies represent these fasting concentrations. Their comparisons provide information on the variation of the concentration of each adduct over the 3 weeks, independent of the breakfast tested since the measurements were pre-prandial.

The fasting free CML concentrations in plasma did not significantly differ from one week to another (*p* = 0.424), and a mean concentration and standard error of 61.6 ± 3.0 nM (*n* = 60) were calculated (Figure 2A). When the fasting free CEL data of the 3 time points were combined (*n* = 60), the mean plasma concentration was 59.0 ± 2.2 nM (Figure 2D). However, unlike CML, a significant difference was observed between the 3 fasting blood samples (*p* < 0.001). Free lysine values were not significantly different (*p* = 0.288) among the 3 “time 0” samples which had a mean concentration of 218.6 ± 6.0 nM (*n* = 60) (Figure 2G). Nor were there any differences in sRAGE concentrations among the 3 time points, and the mean concentration was 1259.8 ± 46.6 pg/mL (*n* = 60) (Figure 2J).

**Figure 3:**
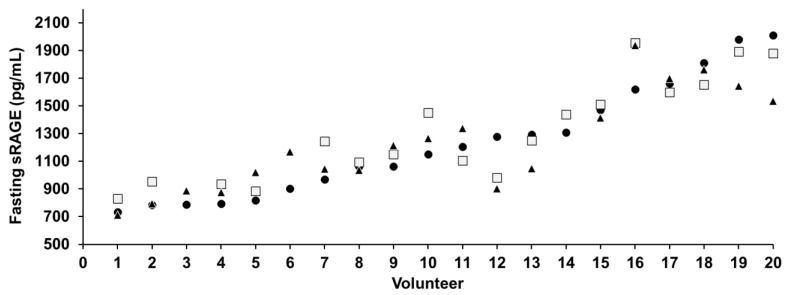
Distribution of fasting plasma sRAGE levels among the 20 volunteers prior to the 3 different interventions (● time 0, □ +7 days, ▲ +14 days).

The distribution of the sRAGE concentration among the 20 healthy volunteers is presented in Figure 3. This figure shows not only that sRAGE ranged from 711 to 2010 pg/mL but also, and perhaps more importantly, that each individual had an almost constant fasting concentration of sRAGE over the 3-week experiment. In other words, people in the lowest (710–969 pg/mL) or highest (1308–2010 pg/mL) tertiles of fasting sRAGE concentrations consistently presented in their respective tertile, regardless of the date of blood collection. This trend was not observed for any of the other 3 adducts quantified in this study. The inter-week relative standard deviation of the mean for each individual (or intra-individual variation for fasting sRAGE) was relatively low (3 to 19%) compared with those for free CML, CEL, and lysine (3–66%, 8–52%, and 2–28%, respectively).

Among the 4 adducts measured in the plasma of the 20 volunteers before breakfast, only free CEL and free lysine were found at higher mean concentrations in male compared with female volunteers (Figure 2F,I).

The Pearson’s correlation coefficients among pairs of the four plasma adducts at their fasting concentrations were calculated (data not shown), and only fasting free CML showed a significant positive correlation with fasting free CEL (r = 0.556, *p* < 0.001).

#### 3.2.3. Free CML, CEL, Free Lysine, and sRAGE in Human Plasma following Breakfast Intakes

In a similar fashion to plasma concentrations in the rats following gavage, free CML increased in human plasma after the intake of all 3 tested breakfasts (Figure 2A). The relatively low dCML dose in the breakfasts, the small population, and the high variability of free CML concentrations among them probably combined to prevent a statistically significant trend over time being found for this adduct. However, the pattern of postprandial kinetics appears very similar to that observed in the rat study, with a maximum plasmatic free CML at 90 min for 2 out of the 3 breakfasts tested. No significant effects of the breakfast type were observed on the kinetics (Figure 2A) or the iAUC_freeCML 0–120 min_ of the free CML concentration in plasma (Figure 2B). When the 3 breakfasts’ kinetics were analyzed as a single group, the mean maximum postprandial concentration of free CML was 71.9 ± 3.0 (range: 26.3 to 139.1) nM, which corresponds to a mean increase of 17% from baseline.

The kinetics of free CEL in human plasma after consumption of the 3 different breakfasts are presented in Figure 2D. No differences were found in free CEL concentration between fasting and postprandial blood samples, and no classical pattern of nutrient absorption was observed. No differences in iAUC_freeCEL 0–120 min_ were observed between the 3 breakfasts (Figure 2E). Overall, this suggests that the slight variations of free CEL concentration observed are not related to the intake of any of the 3 breakfasts.

The concentration of free lysine in the plasma followed a similar trend after all 3 breakfasts, increasing from 0 to 45 min in the MB-, WB- and GWB-fed volunteers (Figure 2G). From 45 to 120 min, however, different kinetic patterns were observed, and calculation of the iAUC_freelysine 0–120 min_ indicated a significantly lower absorption of lysine after eating the GWB compared with the WB (*p* < 0.016) (Figure 2H).

The changes in plasma sRAGE following each breakfast intake are presented in Figure 2J. Plasma sRAGE concentrations showed a sustained postprandial decrease from 0 to 45 min whatever the breakfast consumed. Although not statistically significant, a decrease compared with the fasting level of 10 to 15% of the mean concentration of sRAGE was observed at 45 min, and concentrations remained more or less at these levels thereafter. The overall pattern of postprandial kinetics of sRAGE in plasma is comparable to those reported by Fotheringham et al. in healthy and diabetic individuals [11]. There were no breakfast-specific effects on sRAGE when individual time points of the kinetics (Figure 2J) or iAUC were compared (Figure 2K).

## 4. Discussion

This study is the first, to our knowledge, to evaluate the fasting and postprandial concentration of free CML in both rats and humans using robust and validated LC-MS/MS analytical methods. The description of the kinetics of free CML and related adducts in blood after a single ingestion of different forms of well-controlled doses of dCML casts some much-needed light upon their systemic bioavailability and elimination.

Rodent assays for studying human digestion of glycated proteins are common, but their limitations are well known. Hence, we also aimed to compare rat and human fasting concentrations of free CML in plasma, in addition to its postprandial concentrations.

The fasting plasma concentrations of free CML in rats were 7 times higher than we observed in humans (437 ± 283 vs. 62 ± 3 nM, respectively), a magnitude very similar to that found in the literature. One of our previous rat studies reported a fasting plasma CML concentration of 588 nM [8], and two independent human studies measured 78 (66–99) and 74 ± 6 nM [12,13]. Although the present study does not permit the identification of the origin of this difference in baseline plasmatic free CML concentrations, we can offer two hypotheses. First, although all previously conducted studies have shown no relationship between dCML intake and fasting plasma free CML [8,12], it may be that the much higher, lifelong exposure to dCML of laboratory rats compared with humans could permanently increase their baseline plasma concentration of free CML. The dCML accumulated in certain organs may be partly released during fasting and affect the baseline level of free CML in the circulation [14]. We calculate that with a daily intake of 20 g of standard chow, rats weighing 250 g are exposed to approximately 1450 µg CML/kg/day [15] compared to only 83 µg CML/kg/day in adult humans [3]. The second hypothesis involves the turnover of proteins in mammals. Free CML found in blood derives not only from the digestion of dietary glycated proteins (and absorption of dCML) but also from the in vivo catabolism of glycated proteins. The faster protein turnover in rats compared with humans [16], despite the similar level of CML measured in proteins in the two species [17], may explain the higher baseline plasma-free CML concentration in rats.

In addition to free CML, protein-bound CML was also quantified in the plasma of rats in receipt of different forms of dCML. This revealed plasmatic protein-bound CML at similar concentrations to free CML in fasting animals (391 ± 93 vs. 437 ± 283 nM, respectively). It also showed that the concentration of protein-bound CML varied little between animals and, more importantly, did not change significantly after an oral exposure to dCML in whatever form it was administrated (free or protein-bound). Our previous rat study [8] and clinical intervention [18], as well as the recent CODAM study [12], also concluded that dCML has no impact on the concentration of protein-bound CML in the circulation and that the latter very likely derives only from in vivo glycation of circulating proteins.

The plasma kinetics of free CML after four different single oral doses of dCML in rats, or after three different breakfast meals in humans, revealed comparable but slightly different patterns of absorption and elimination. Firstly, a comparison of the kinetics after gavage with native free dCML or its isotope indicated that a single, high dose of native dCML is sufficiently accurate to assess uptake and elimination of dCML in rats. Under the experimental conditions described, it appeared that the post-gavage kinetics of plasmatic free CML were unaffected by endogenous sources of free CML. We concluded that changes in plasmatic free CML relative to the fasting concentration were due solely to the exogenous dose of CML (i.e., dCML) in the gavage.

Paul-André Finot, a pioneer in the study of the metabolic transit of chemically modified amino acids, highlighted the difference in the absorption of free and protein-bound glycation products [19]. Our comparative animal study revealed, in line with Finot’s observations, that the absorption of dCML followed different kinetic profiles depending on the form of CML ingested (as free or protein-bound CML or CML administered with a protein). On the other hand, the total absorption of dCML was not affected by the form in which it was administered (non-significant difference of iAUC among the four conditions tested). The different kinetics of plasmatic free CML may reflect a slower release of dCML when it is ingested in protein-bound form (CML-BSA) and hint at a competition for the transport of dCML with other amino acids from the gut to the circulation (free CML + BSA). Such animal tests and food model systems are useful for understanding the metabolic transit of dCML. Still, it is important to remember that this approach uses much higher doses than those to which humans are generally exposed.

The second part of the current study aimed to observe the postprandial plasma-free CML response after the intake of three classic breakfasts: a Mediterranean-inspired breakfast (MB), a Western-inspired breakfast (WB), and a Grilled Western-inspired Breakfast (GWB). According to the chemical analysis of the three meals, the intake of dCML ranged from 2.02 ± 0.05 to 2.73 ± 0.04 mg/meal (MB and GWB, respectively). These levels of dietary exposure accord with the daily exposure calculated in our previous intervention study (2.2 ± 0.9 and 5.4 ± 2.3 mg/day for a “steam” and a “standard” French diet) [3] and with that estimated in other epidemiological and clinical studies (2.1 to 4.2 mg/day [12]; 2.6 to 4.9 mg/day [20]). The range of CML exposures, as well as the absolute exposures in both our intervention study and other human studies, are very much lower than those tested in animal studies (including ours).

When exposure to dCML is expressed in proportion to body weight, the twenty healthy volunteers received between 31 ± 1 and 42 ± 1 µg CML/kg BW in one or other of the 3 different breakfasts. In contrast, we tested an average of 1570 ± 150 µg CML/kg BW in rats receiving a single oral gavage. Despite this large difference in exposure, the kinetics of postprandial absorption of free CML in humans appeared to be similar to that observed in rats (i.e., a postprandial peak of plasmatic free CML at 90 to 120 min). As expected, the differences in dCML exposure among the 3 breakfasts were too small to yield any significant difference in the iAUC_freeCML 0–120 min_ of the free CML concentration in plasma. Our previous nutritional intervention trial (ICARE clinical study) [18], as well as the more recent cross-sectional CODAM study [12], also report only a weak or non-significant direct relationship between dCML and free CML in fasting plasma or urine. The relatively low dose of dCML in a regular meal offers a likely explanation of the weakness of the association between ingested dCML and free CML in plasma (plasmatic free CML increased by at most 17% from baseline in our study) and may mean that significant relationships between intake and circulating concentrations of CML are observed only after controlling for several factors pertinent to an individual’s metabolism. This appears to be the baseline case, fasting levels of plasmatic free CML: the CODAM study reported a significant association between dCML and fasting plasmatic free CML only after controlling for age, sex, glucose metabolism, and waist circumference. This finding would indirectly support our current and previous observations that recent dCML intake affects postprandial plasmatic free CML but not baseline, fasting free CML levels [8].

Much less research exists on CEL, even though this AGE is also present in food at a level close to CML. The 3 breakfasts studied here contained 1.66 ± 0.03 to 3.39 ± 0.05 mg of dCEL/meal compared to 2.02 ± 0.05 to 2.73 ± 0.04 mg of dCML/meal. Despite the fact that the study volunteers were exposed to dCEL, no postprandial increase of free CEL was observed whatever the breakfast tested. This absence of a postprandial increase in circulating free CEL agrees with recent observations by Perkins et al. in healthy, obese adults [13]. As seen for free CML, the mean fasting free CEL concentrations were comparable: 59.1 ± 10.6 nM among the 20 healthy adults in the current study and 55 ± 5 nM among the 10 healthy obese adults studied by Perkins et al. [13].

The only difference in the chemical structures of CML and CEL is an extra methyl group in CEL. To our knowledge, no significant difference in absorption of these two AGEs by intestinal cells has been described, so the most reasonable hypothesis for this difference in postprandial kinetics is a different metabolic transit of these dAGEs [7]. It is worth mentioning that fasting-free CML was positively correlated with fasting-free CEL in our human study. Could this indicate a common endogenous origin of the basal level of these 2 free AGEs? In this case, could they be two circulating biomarkers of carbonyl stress? This theory remains to be investigated.

For the first time, to our knowledge, we report that fasting-free CEL was at a lower concentration in women (though confirmation of this observation in a larger cohort is required). This gender difference was also observed for fasting free lysine but not for fasting free CML. Even though significant differences in most circulating free amino acids between men and women were reported many years ago, no clear biological explanation has yet been given [21].

The effects of the Maillard reaction on the nutritional quality of food proteins have been widely studied, the loss of lysine being a major concern for nutritionists. This essential amino acid is one of the main targets of glycation, and its transformation into CML, CEL, and other AGEs results in chemically modified lysine, which is unavailable for protein anabolism after ingestion (also known as “blocked lysine”). Animal experiments [22] and clinical studies [23] conducted with exaggeratedly glycated proteins (>50% blocked lysine) have reported reduced postprandial plasma-free lysine responses. However, when normally processed foods are compared in a clinical study, such as pasteurized and ultra-high temperature processed milk, no significant difference is observed in postprandial plasma lysine concentrations [24]. Our clinical study was not designed to compare 3 breakfasts with an equal quantity of proteins (and hence lysine) (Table 1), and any comparison of the postprandial plasmatic free lysine kinetics must be made with this in mind (Figure 2G,H). Nevertheless, the WB and GWB contained similar amounts of intact lysine, yet the latter exhibited lower postprandial free lysine concentrations (*p* = 0.016). While our study was not designed specifically to address this question, this result suggests that when the sandwich (bread, cheese, and ham) was toasted for 4 min, the heat treatment of the protein may have reduced lysine uptake. A decreased protein digestibility has been described for thermally processed foods tested in in vitro digestive model systems, but is not consistently found in clinical studies [5].

In addition to the nutritional issue of the assimilation of the essential amino acid lysine, the main reason for measuring free lysine in the plasma of our healthy volunteers was to compare the kinetic trends between the uptake of free lysine and its glycated derivatives CML and CEL and confirm that they were indeed similar.

The putative deleterious effects of dCML and other dAGEs are often justified by their affinity for the cell-membrane RAGE receptor and the consequent activation of pro-inflammatory and pro-oxidative pathways. While some studies have reported that protein-bound CML is unable to bind RAGE and induce a pro-inflammatory response [25], others suggest that this type of glycated protein is an important ligand for RAGE, while free CML is not [26]. The binding of protein-bound CML and other glycated proteins to RAGE and the subsequent activation of RAGE signaling and its associated pathological consequences are nevertheless widely accepted as a key biological mechanism by which dAGEs may affect health. Other studies have not only confirmed that free CML and CEL do not bind to RAGE, but also report that protein-bound CML is only a weak ligand compared to protein-bound MG-H1 (Methylglyoxal-derived hydroimidazolone 1) [27]. Considering that free AGEs (i.e., modified amino acids of low molecular weight) are unlikely to be RAGE ligands, it is difficult to understand how they can be involved in RAGE signaling after intestinal absorption. The discovery of soluble isoforms of RAGE (sRAGE including es- and c-RAGE) complexified the study of the so-called AGE-RAGE axis [28] but also offered hope for therapy against glycation-related pathologies. It is considered (perhaps simplistically) that sRAGE may act as a decoy receptor for AGEs and thus prevent or reduce the activation of inflammatory and oxidative pathways.

With all this in mind, the current clinical study aimed to evaluate the change in plasma sRAGE after the intake of 3 different breakfasts containing different amounts of CML and CEL. Prior to administration, fasting sRAGE was at an average concentration of 1260 ± 373 pg/mL with no difference between males and females. This absence of a gender difference has already been observed in cohorts of different ages [29,30,31]. Notably, each participant had a unique and remarkably constant level of sRAGE in the blood samples collected over the 3 weeks of the study. Clinical data on the association between fasting sRAGE and serum concentrations of protein-bound AGEs are discordant and scarce, however. Yamagishi et al. found a positive association among 184 non-diabetic Japanese subjects [31], while De Courten et al. reported a moderate negative association among 20 non-diabetic, overweight Australian subjects [20]. Our current clinical study tested the association between sRAGE and free CML and CEL levels at baseline and up to 2 h postprandial, and no correlation was found.

Temporal fluctuations of sRAGE in humans remain poorly understood. Synthesizing the limited data available is further complicated by the fact that they derive from type 1 or 2 diabetic patients, from obese and healthy subjects [11,13,32]. Despite the heterogeneity of these populations, a common trend of daily circulating sRAGE is nevertheless evident, with its production higher following an overnight fast [32] and decreasing after breakfast [11,13]. The current study confirms that plasma sRAGE concentrations decreased after breakfast, while we additionally observed here that this was independent of the dAGE contents of the meals. As described in type 1 diabetic patients by Miranda et al., no statistically significant inverse correlation was found between sRAGE and free CML or CEL among the healthy participants in our study. Among the free AGEs measured by Miranda et al., only the overnight decrease in free MG-H1 was associated with the concurrent increase in sRAGE levels. Among other things, the authors suggested that the higher affinity for RAGE of MG-H1 compared to CML and CEL could be the reason for this only inverse correlation. However, we were unable to find a study demonstrating that a free AGE could bind to RAGE (either soluble or membrane-bound), and an elimination of MG-H1 and other free AGEs by a mechanism of sequestration by sRAGE has yet to be demonstrated.

The inverse fluctuation of circulating sRAGE and free AGEs observed here, and the more or less significant inverse correlations observed by the pioneering study of Miranda et al. are far from proof that sRAGE traps circulating free AGEs and eliminates them. Since no control group without breakfast intake was included in our clinical study, the direct effect of food intake on circulating sRAGE concentration remains to be proven. This also applies to the association of sRAGE and free AGEs following an overnight fast [32].

## 5. Conclusion

In conclusion, research on the time-dependent modulation of sRAGE and the metabolic transit of dAGEs and other MRPs merits further investigation. While we have shed a little more light on the relationship between free AGEs and sRAGE in plasma with this study, the biological mechanisms by which dietary and other environmental factors affect their circulating concentrations require further elucidation.

## Figures and Tables

**Table 1:** Characteristics of the three breakfasts.

	Unit	MB	WB	GWB	*p*-Value		
					MB × WB	MB × GWB	WB × GWB
Fat content	% (g/100 g)	3.8 ± 0.4	2.3 ± 0.2	2.7 ± 0.3	<0.001	<0.001	<0.001
Calories from fat	kcal/100 g	34 ± 3.4	21 ± 2.1	24 ± 2.4	<0.001	<0.001	0.002
Saturated fatty acids	% of total fatty acids	52.9 ± 5.2	57.5 ± 5.7	56.5 ± 5.7	0.029	0.108	0.836
Monounsaturated fatty acids	% of total fatty acids	36.1 ± 3.6	32.6 ± 3.3	33.2 ± 3.3	0.005	0.025	0.843
Polyunsaturated fatty acids	% of total fatty acids	10.2 ± 0.1	9.2 ± 0.9	9.6 ± 1.0	<0.001	0.047	0.244
Trans fat	% (g/100 g)	0.80 ± 0.08	0.7 ± 0.07	0.7 ± 0.07	<0.001	<0.001	>0.999
Carbohydrates (including sugars and fibers)	% (g/100 g)	18.3 ± 1.8	15.0 ± 1.5	16.5 ± 1.6	<0.001	0.003	0.015
Total sugars	% (g/100 g)	3.0 ± 0.3	2.3 ± 0.2	2.8 ± 0.3	<0.001	0.059	<0.001
Total dietary fibers	% (g/100 g)	2.6 ± 0.3	2.2 ± 0.2	2.1 ± 0.2	<0.001	<0.001	0.385
Crude fibers	% (g/100 g)	1.4 ± 0.1	1.1 ± 0.1	0.9 ± 0.1	<0.001	<0.001	<0.001
Protein (N × 6.25)	% (g/100 g)	4.1 ± 0.4	6.1 ± 0.6	6.0 ± 0.6	<0.001	<0.001	0.829
Total breakfast ingested (*n* = 20)	g	389.1 ± 9.1	437.1 ± 6.1	413.6 ± 5.5	<0.001	<0.001	<0.001
Total CML	mg/100 g	0.52 ± 0.02	0.46 ± 0.01	0.66 ± 0.01	<0.001	<0.001	<0.001
	mg/breakfast	2.02 ± 0.05	2.03 ± 0.03	2.73 ± 0.04	0.720	<0.001	<0.001
Total CEL	mg/100 g	0.47 ± 0.01	0.38 ± 0.01	0.82 ± 0.02	<0.001	<0.001	<0.001
	mg/breakfast	1.83 ± 0.05	1.66 ± 0.03	3.39 ± 0.05	<0.001	<0.001	<0.001
Total lysine	mg/100 g	472 ± 7	547 ± 1	681 ± 37	<0.001	<0.001	<0.001
	mg/breakfast	1836 ± 43	2391 ± 33	2817 ± 46	<0.001	<0.001	<0.001
Ash content	% (g/100 g)	1.10 ± 0.01	0.70 ± 0.01	1.00 ± 0.01	<0.001	<0.001	<0.001
Moisture content	% (g/100 g)	72.7 ± 7.2	75.9 ± 7.6	73.8 ± 7.4	0.365	0.886	0.644
Caloric value	kcal/100 g	124 ± 12	105 ± 10	114 ± 11	<0.001	0.021	0.042
Iron	mg/kg	31.2 ± 3.1	31.5 ± 3.1	40.6 ± 4.1	0.960	<0.001	<0.001
Sodium	g/kg	2.3 ± 0.2	2.0 ± 0.2	1.9 ± 0.2	<0.001	<0.001	0.262
Calcium	% (g/100 g)	0.10 ± 0.01	0.09 ± 0.01	0.07 ± 0.01	0.007	<0.001	<0.001
Vitamin C	mg/100 g	2.2 ± 0.2	1.5 ± 0.1	0.9 ± 0.1	<0.001	<0.001	<0.001
Vitamin E	µg/100 g	385 ± 38	127 ± 12	96 ± 9	<0.001	<0.001	<0.001

CEL: carboxyethyllysine; CML: *N^Ɛ^*-carboxymethyllysine; GWB: Grilled Western-inspired Breakfast; MB: Mediterranean-inspired breakfast; WB: Western-inspired breakfast. Data represent mean ± SD. Statistical significance among mean values was evaluated within each line, and reported *p* values were calculated considering *α* = 0.05. Standard methods are indicated in the Supplemental Table S1.

## Supplementary Materials

The following supporting information can be downloaded at: https://www.mdpi.com/article/10.3390/nu14091890/s1, Table S1: List of standard methods applied for analysis of calories, fat, carbohydrates, protein, ash, moisture, minerals, and vitamins.
