# Supplementary material for: Plasma Levels of Free NƐ-Carboxymethyllysine (CML) after Different Oral Doses of CML in Rats and after the Intake of Different Breakfasts in Humans: Postprandial Plasma Level of sRAGE in Humans
Source: Nutrients. 2022 Apr 30;14(9):1890. doi: 10.3390/nu14091890 (PMC9101122; doi:10.3390/nu14091890)
Supplement: Supplementary file 1 [file nutrients-14-01890-s001.zip › nutrients-1671753-supplementary.pdf]

Supplementary Information (SI)

Article

Plasma Levels of Free *N*$^{\varepsilon}$-Carboxymethyllysine (CML) after Different Oral Doses of CML in Rats and after the Intake of Different Breakfasts in Humans: Postprandial Plasma Level of sRAGE in Humans

Cynthia Helou [1,†], Matheus Thomaz Nogueira Silva Lima [2,†], Céline Niquet-Leridon [3], Philippe Jacolot [3], Eric Boulanger [2], Florian Delguste [2], Axel Guilbaud [2], Michael Genin [4], Pauline M. Anton [3], Carine Delayre-Orthez [3], Tatiana Papazian [1], Michael Howsam [2] and Frédéric J. Tessier [2,*]

[1] Department of Nutrition, Faculty of Pharmacy, Saint Joseph University of Beirut, Beirut 1004 2020, Lebanon; cynthia.helou@usj.edu.lb (C.H.); tatiana.wahanian@usj.edu.lb (T.P.)
[2] U1167—RID—AGE—Facteurs de Risque et Déterminants Moléculaires des Maladies Liées au Vieillissement, Institut Pasteur de Lille, University Lille, Inserm, CHU Lille, F-59000 Lille, France; matheusthomaz.nogueirasilvalima@univ-lille.fr (M.T.N.S.L.); eric.boulanger@univ-lille.fr (E.B.); florian.delguste@gmail.com (F.D.); axel.guilbaud@gmail.com (A.G.); michael.howsam@univ-lille.fr (M.H.)
[3] ULR 7519, Equipe PETALES, Institut Polytechnique UniLaSalle, Université d'Artois, F-60026 Beauvais, France; celine.leridon@unilasalle.fr (C.N.-L.); philipe.jacolot@unilasalle.fr (P.J.); pauline.anton@unilasalle.fr (P.M.A.); carine.delayre@unilasalle.fr (C.D.-O.)
[4] ULR 2694-METRICS: Évaluation des Technologies de Santé et des Pratiques Médicales, University Lille, Inserm, CHU Lille, F-59000 Lille, France; michael.genin@univ-lille.fr
* Correspondence: frederic.tessier@univ-lille.fr; Tel.: +33-(0)3-2062-3561
† These authors contributed equally to this work.

This PDF file includes:

Table S1. List of standard methods applied for analysis of calories, fat, carbohydrates, protein, ash, moisture, minerals, and vitamins.

	**Method**
**Fat content**	AOAC 945.48, 933.05 & 963.15
**Calories from fat**	For calorie conversion, calorie values were calculated based on the total grams of fat equivalent to 9 kcal/g.
**Saturated fatty acids**	Fatty Acid Profile (saturated, unsaturated, trans): Hoteit, M., Zoghbi, E., Rady, A., Shankiti, I., & Al-Jawaldeh, A. (2021). Fatty acids quality in middle eastern traditional dishes, arabic sweets and market foods frequently consumed in Lebanon. *Nutrients*, *13*(7). https://doi.org/10.3390/nu13072462
**Monounsaturated fatty acids**	
**Polyunsaturated fatty acids**	
**Trans fat**	
**Carbohydrates (including sugars and fibers)**	Carbohydrates were calculated by subtracting the sum of the percentages of the measured weights of fat, protein, moisture, and ash from the total weight (100g)
**Total sugars**	AOAC 906.03, 930.36 and 975.15
**Total dietary fibers**	AOAC 985.29
**Crude fibers**	AOAC 962.09
**Protein (N x 6.25)**	AOAC 991.20-23 crude protein was calculated through the multiplication of total Kjeldahl nitrogen by 6.25
**Ash content**	Ash content is determined by incineration in an Isotemp muffle furnace at 550°C overnight
**Moisture content**	The food samples were heated to 105°C (Fisher Isotemp vacuum oven) and the measurement of moisture content was based on the loss of weight of the sample.
**Caloric value**	For calorie conversion, calorie values were calculated based on the total grams of protein, carbohydrate, and fat equivalent of 4, 4, and 9 kcal/g, respectively.
**Iron**	AOAC 985.35
**Sodium**	AOAC 985.35
**Calcium**	AOAC 985.35
**Vitamin C**	AOAC 967.21-1968
**Vitamin E**	NF-EN 12822
